# Sodium-glucose cotransporter 2 inhibitors in patients with type 2 diabetes and myocardial infarction undergoing percutaneous coronary intervention: A systematic review and meta-analysis^☆^

**DOI:** 10.1016/j.ajpc.2024.100927

**Published:** 2024-12-31

**Authors:** Huzaifa Ul Haq Ansari, Muhammad Ammar Samad, Eman Mahboob, Eeshal Zulfiqar, Shurjeel Uddin Qazi, Areeba Ahsan, Mushood Ahmed, Faizan Ahmed, Raheel Ahmed, Shafaqat Ali, Mahboob Alam, Jamal S. Rana, Gregg C. Fonarow

**Affiliations:** a Dow University of Health Sciences, Karachi, Pakistan; b Foundation University Medical College, Islamabad, Pakistan; c Rawalpindi Medical University, Rawalpindi, Pakistan; d Division of Cardiology, Duke University Hospital, Durham, NC, USA; e Department of Cardiology, Royal Brompton Hospital, London, UK; f National Heart and Lung Institute, Imperial College London, UK; g Department of Cardiology, Louisiana State University, Shreveport, USA; h Department of Cardiology, Baylor College of Medicine, Houston, TX, USA; i Division of Cardiology, Kaiser Permanente Northern California, Oakland, CA, USA; j Division of Research, Kaiser Permanente Northern California, Oakland, CA, USA; k Ahmanson-UCLA Cardiomyopathy Center, Division of Cardiology, University of California Los Angeles, Los Angeles, CA, USA

**Keywords:** Sodium-glucose co-transporter 2 inhibitors, Type 2 diabetes, Myocardial infarction, Percutaneous coronary intervention

## Abstract

**Background:**

Sodium-glucose cotransporter 2 inhibitors (SGLT2i) have shown benefits in improving cardiovascular (CV) outcomes in patients with heart failure (HF) and may mitigate symptom progression in myocardial infarction (MI). However, their effectiveness in patients with type 2 diabetes and MI undergoing percutaneous coronary intervention (PCI) is unclear.

**Methods:**

To identify eligible studies, a comprehensive search of electronic databases, PubMed, Cochrane Library, Scopus and Embase, was conducted from inception until May 2024. Results were presented as risk ratios (RR) and their corresponding 95 % confidence intervals (CIs).

**Results:**

Our analysis included 8 observational studies comprising 24,229 patients. The results indicated that SGLT2i with PCI was associated with a significantly reduced risk of all-cause death (RR=0.61; 95 % CI=0.54 to 0.68), CV death (RR=0.46; 95 % CI=0.22 to 0.94), major adverse cardiovascular events (RR=0.80;95 % CI: 0.66 to 0.96), HF-related hospitalizations (RR=0.63; 95 % CI=0.44 to 0.90), stroke (RR=0.77; 95 % CI: 0.62 to 0.96) and acute kidney injury (RR=0.46; 95 % CI: 0.25 to 0.84) compared to PCI without SGLT2i use. However, the risk of revascularization remained comparable between the groups.

**Conclusion:**

Our study demonstrates that SGLT2i with PCI in patients with type 2 diabetes and MI are associated with improved CV outcomes compared to PCI without SGLT2i use. Randomized controlled trials are required to confirm the improvement in outcomes with SGLT2i therapy combined with PCI in patients with MI and diabetes.

## Graphical abstract

**Figure:**
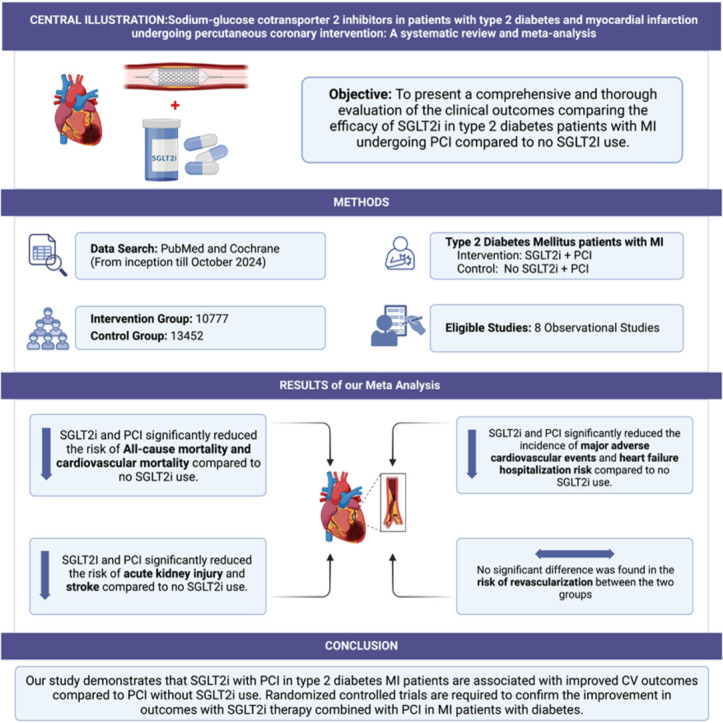

## 1 Introduction

Acute myocardial infarction (AMI) is a major global cause of morbidity and mortality [1,2]. Percutaneous coronary intervention (PCI) is a commonly used, minimally invasive procedure in the treatment of AMI [3]. While effective, patients with AMI undergoing PCI are at a considerable risk of developing acute kidney injury (AKI), recurrent cardiovascular events, and heart failure [4,5]. Notably, contrast-induced acute kidney injury (CI-AKI) occurs in 1.3 to 33.3 % of people undergoing PCI and is significantly associated with in-hospital mortality [6].

Sodium-glucose cotransporter 2 inhibitors (SGLT2i) are oral hypoglycemic agents that reduce blood glucose levels through the inhibition of renal tubular reabsorption of glucose [7]. Other than their effects on glycemic control, recent research has also shown the benefits of SGLT2i in improving cardiovascular (CV) and renal outcomes in patients with heart failure [8,9]. These benefits are hypothesized to result from their kidney-mediated natriuretic effects, improved blood flow regulation, reduced endothelial dysfunction, as well as their role in reducing infarct size and improving left ventricular function post-AMI, thus preventing progression to HF [10,11]. Recent literature on the cardioprotective function of SGLT2i proposes that it may be due to the induction of autophagy. Specifically, empagliflozin (EMPA) has been shown to suppress autosis by inhibiting the Na+/*H*+ exchanger 1 (NHE1) in cardiomyocytes, optimizing autophagic flux and reducing myocardial ischemic injury. This leads to improvements in LV function post-AMI [12]. Moreover, SGLT2i have been observed to induce a cardiac metabolic shift toward ketone utilization, which increases circulating ketone levels. This shift is associated with improved myocardial efficiency and reduced oxygen consumption that is crucial for preserving cardiac function in ischemic conditions, particularly after PCI [13,14].

However, there remains a lack of evidence specifically addressing their effectiveness in patients with type-2 diabetes and MI undergoing PCI, warranting a meta-analysis with enhanced statistical power. Therefore, we aim to evaluate whether SGLT2i combined with PCI improves clinical outcomes in compared to PCI alone.

## 2 Methods

The current systematic review and meta-analysis is reported according to Preferred reporting items for systematic review and meta-analyses (PRISMA) guidelines [15].

### 2.1 Data sources and search strategy

To retrieve all relevant articles, a literature search was conducted on PubMed, Cochrane Library, Scopus and Embase from the inception until May 2024 using the following keywords with their associated MeSH terms: “(Sodium-glucose co-transporter inhibitor-2 OR SGLT2 inhibitors OR empagliflozin OR dapagliflozin OR canagliflozin OR bexagliflozin) AND (myocardial infarction OR MI) AND (percutaneous coronary intervention OR PCI). The detailed search strategies are reported in **Table S1**. In addition, we thoroughly searched the reference lists of the retrieved articles, past review articles, and meta-analyses, to find any relevant studies that may have been missed in the search.

### 2.2 Study selection and bias assessment

The articles were selected for inclusion if they fulfilled the following eligibility requirements: [1] randomized Control Trials (RCTs) or observational cohorts, [2] the patients enrolled in the studies were ≥ than 18 years of age, and [3] compared outcomes of SGLT2i in patients with type-2 diabetes with MI undergoing PCI to the outcomes when no SGLT2i are used post-PCI. All case reports, review articles, observational studies, and studies on non-human subjects were excluded from our literature review.

The articles retrieved from the systematic search were exported to the EndNote Reference Library X7 software where duplicates were removed. The remaining articles were carefully assessed by two independent reviewers (Q.S.U and H.U.H.A), and only those trials that met the previously defined criteria were selected. All studies were initially short-listed based on title and abstract, after which the full article was reviewed to affirm relevance. All discrepancies were resolved by a third reviewer (A.S).

The risk of bias assessment of the observational studies was performed using the Cochrane Risk of Bias in Nonrandomized Studies - of Interventions (ROBINS-I) tool [16]. The ROBINS-I tool uses seven domains to determine overall bias in each non-randomized clinical trial. Studies were classified as having low, moderate, serious, or critical risk of bias. Studies that had information missing in one or more domains were classified as NI (no information). Sensitivity analyses were conducted using the leave-one-out approach to investigate the impact of individual studies on the overall results.

### 2.3 Data extraction and outcomes

The extracted data included patient demographics as well as outcome information. The demographics of the patients included sample size, age of patients, body mass index, and history of smoking, dyslipidemia and hypertension.

The primary outcomes included all-cause death and cardiovascular death. The secondary outcomes included major adverse cardiovascular events (MACE), hospitalization due to heart failure (HHF), revascularization, acute kidney injury (AKI) and stroke. The criteria used for reporting MACE in each study is mentioned in **Table S2**.

### 2.4 Statistical analysis

All statistical analysis was performed on Review Manager (Version 5.4.1, Copenhagen: The Nordic Cochrane Centre, The Cochrane Collaboration, 2014). The risk ratios were calculated to present pooled effect sizes along with corresponding 95 % confidence intervals (CIs). Forest plots were generated for a graphical representation of results. The Higgins (I²) statistic was used to evaluate heterogeneity and a value of 25 %−50 % was considered mild, 50 %−75 % as moderate, and >75 % as severe heterogeneity. A p-value of less than 0.05 was considered significant.

## 3 Results

### 3.1 Search results

**Fig. 1:**
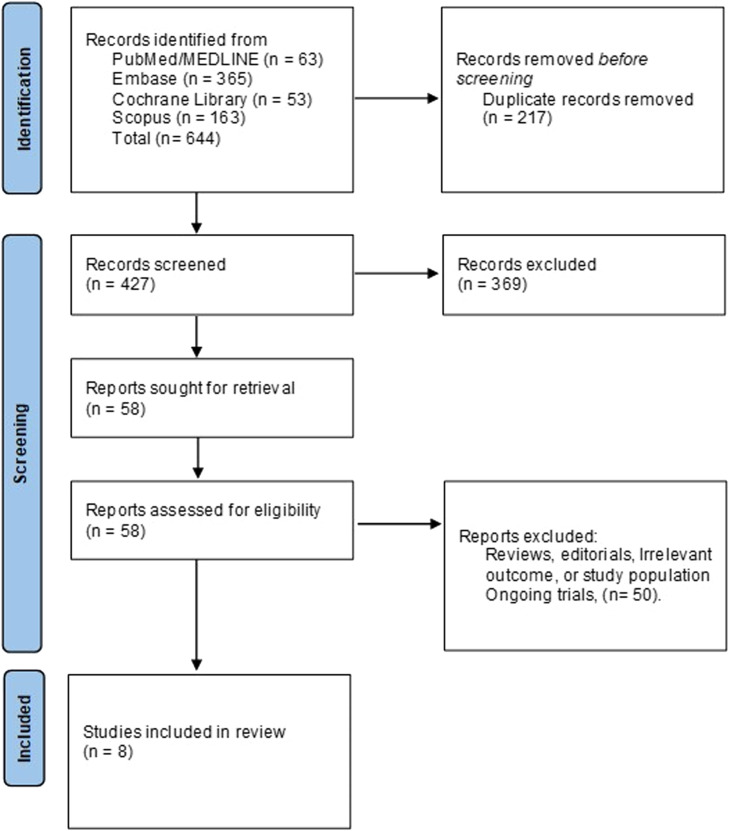
PRISMA flowchart showing the screening and study selection process.

An initial search of the databases yielded 644 records. After removing duplicates, 427 unique records were subjected to title and abstract screening. This process identified 58 potentially eligible reports retrieved for full-text assessment based on the eligibility criteria. Eight studies met the predefined inclusion criteria and were included in the final analysis. The details of screening and the study selection process are provided in Fig. 1.

### 3.2 Study characteristics and risk of bias

A total of 8 studies were included; All of the studies were retrospective observational studies [[17], [18], [19], [20], [21], [22], [23], [24]] published from 2022 to 2024. A total of 24,229 patients were included in our meta-analysis. SGLT2i was administered in 10,777 patients while 13,452 patients received placebo. The median age of patients ranged from 55 to 72 years. Male patients constituted >50 % of the participants in each trial. All studies had a follow-up duration ranging from 6 months to 2 years. The details of the baseline characteristics are provided in Table 1.

**Table 1:** Baseline characteristics of included studies and patients.

Author	Year	Timing of study	Study design	Sample size		Follow-up	Age-median (IQR) or mean± SD		Males-n (%)		BMI-median (IQR) or mean± SD		Hypertension, n (%)		Dyslipidaemia-n (%)		Smoking-n (%)		STEMI-n (%)	
				**SGLT2i**	**Control**		**SGLT2i**	**Control**	**SGLT2i**	**control**	**SGLT2i**	**Control**	**SGLT2i**	**Control**	**SGLT2i**	**Control**	**SGLT2i**	**Control**	**SGLT2i**	**Control**
**Paolisso**	**2022**	January 2018 and November 2021	retrospective	111	535	24 months	66 [59–73]	72 [62–80]	90 (81.1)	405 (75.7)	27.1 [24.6–30]	27.7 [25–31.4]	98 (88.3)	443 (82.8)	90 (81.1)	418 (78.1)	67 (60.4)	303 (56.6)	52 (46.8)	257 (48)
**Cai**	**2024**	January 2017 to August 2021	retrospective	278	1561	2 years	62 ± 13.2	65.1 ± 14.0	212 (76.3 %)	1146 (73.4 %)	25.7 ± 3.8	24.2 ± 3.6	216 (77.7 %)	1035 (66.3 %)	NR	NR	120 (43.2 %)	739 (47.3 %)	171 (61.5 %)	982 (62.9 %)
**Kwon**	**2023**	January 2013 to August 2018	retrospective	938	1876	2.1 years	56.4 ± 11.3	57.6 ± 11.3	769 (82.0)	1482 (79.0)	NR	NR	699 (74.5)	1398 (74.5)	591 (63.0)	1182 (63.0)	NR	NR	550 (58.6)	1137 (60.6)
**Kültürsay**	**2024**	2021 and 2022	retrospective	130	165	–	58.5 ± 9.6	61.4 ± 9.0	99 (76.1)	103 (62.4)	NR	NR	110 (84.6)	99 (60)	76 (58.5)	61 (37)	73 (56.2)	97 (58.8)	60 (46.2)	35 (21.2)
**Chen**	**2024**	June 2020 and September 2023	retrospective	63	63	3 months	56 (54–59)	56 (51–59)	40 (64)	34 (54)	22.71 ± 1.99	22.63 ± 1.69	27 (42.86)	25 (39.68)	NR	NR	NR	NR	63 (100)	63 (100)
**Kim**	**2024**	January 2014 and December 2019	retrospective	4610	4610	–	62.3 ± 10.58	62.24 ± 10.94	3494 (75.79)	3485 (75.6)	26.58 ± 3.47	26.48 ± 3.52	4021 (87.22)	4010 (86.98)	4454 (96.62)	4424 (95.97)	673 (24.42)	699 (25.36)	NR	NR
**Lyu**	**2023**	January 2016 to June 2020	retrospective	537	532	12 months	63.21 ± 11.17	63.74 ± 11.33	358 (66.7)	388 (72.9)	NR	NR	350 (65.1)	344 (64.6)	125 (23.3)	133 (25.0)	254 (47.2)	283 (53.2)	244 (45.5)	241 (45.3)
**Lee**	**2023**	1 May 2016–31 December 2019	retrospective	4110	4110	1.7 years	61.7 ± 11.3	62.3 ± 10.8	3236 (78.73 %)	3265 (79.44 %)	NR	NR	3201 (77.88 %)	3198 (77.81 %)	3402 (82.77 %)	3416 (83.11 %)	NR	NR	NR	NR

SGLT2i: Sodium-Glucose Transport Protein 2 Inhibitors, BMI: body mass index, STEMI: ST-segment elevation myocardial infarction, NR: not reported, n: number.

Of 8 observational studies, two [17,20] were judged to have some concerns due to confounding or deviations from intended interventions. The details are provided in **Figure S1**.

### 3.3 Clinical outcomes

#### 3.3.1 All-cause death

**Fig. 2:**
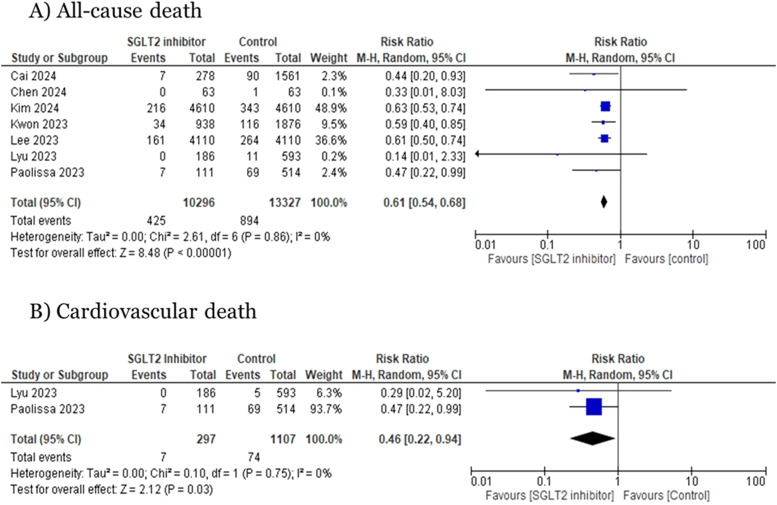
Forest plots for (A) All-cause death and (B) Cardiovascular death.

Seven studies reported data on all-cause mortality. SGLT2i plus PCI was associated with a significant decrease in the risk for all-cause death in comparison to the group receiving no SGLT2i post-PCI strategy (RR=0.61; 95 % CI=0.54, 0.68; *p* < 0.01; I^2^ =0 %, Fig. 2**A**).

#### 3.3.2 Cardiovascular death

Two studies reporting data on death due to cardiovascular causes were analyzed. The pooled analysis highlighted a significant risk reduction associated with SGLT2i plus PCI procedure compared to no SGLT2i use (RR=0.46; 95 % CI=0.22, 0.94; *p* = 0.03; I^2^ =0 %) (Fig. 2**B**).

#### 3.3.3 Major adverse cardiovascular events (MACE)

**Fig. 3:**
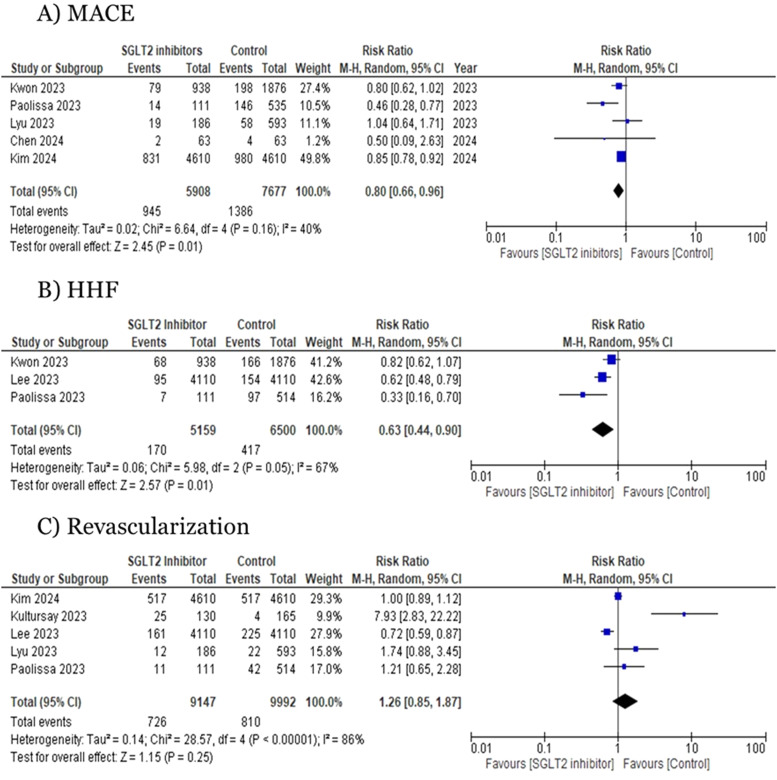
Forest plots for (A) MACE and (B) HHF MACE: major adverse cardiovascular events, HHF: hospitalizations due to heart failure.

Five studies reported data on MACE. SGLT2i plus PCI was associated with a significant decrease in the risk for MACE compared to no SGLT2i use post-PCI (RR=0.80;95 % CI=0.66, 0.96; *p* = 0.01; I^2^=40 %, Fig. 3**A**).

#### 3.3.4 Hospitalization due to heart failure

Three studies reporting on the risk of HHF were analyzed. Pooled analysis showed a significant decrease in the risk of HHF associated with SGLT2i plus PCI compared to no SGLT2i use post-PCI (RR=0.63; 95 % CI=0.44, 0.90; *p* < 0.01; I^2^ =67, Fig. 3**B**). Owing to the high level of heterogeneity observed, a leave-one-out analysis was conducted, during which the study by Paolisso 2023 et al. was excluded. This exclusion substantially reduced the heterogeneity from 67 % to 56 %% and the results remained significant (RR= 0.71, 95 % CI: 0.54 to 0.93, *p* < 0.01, I² =56 %) (Figure S2)

#### 3.3.5 Revascularization

Five studies reporting on the risk of revascularization were analyzed. Pooled analysis showed a non-significant risk association between the two groups (RR=1.26; 95 % CI=0.85, 1.87; *p* = 0.25; I^2^ =86 %, Fig. 3**C**). Owing to the high level of heterogeneity observed, a leave-one-out analysis was conducted, during which the study by Kultursay 2023 was excluded. This exclusion reduced the heterogeneity from 86 % to 75 % and the results remained non-significant (RR = 0.98, 95 % CI: 0.73 to 1.31, *p* = 0.89, I² = 75 %) (Figure S3)

#### 3.3.6 Acute kidney injury

**Fig. 4:**
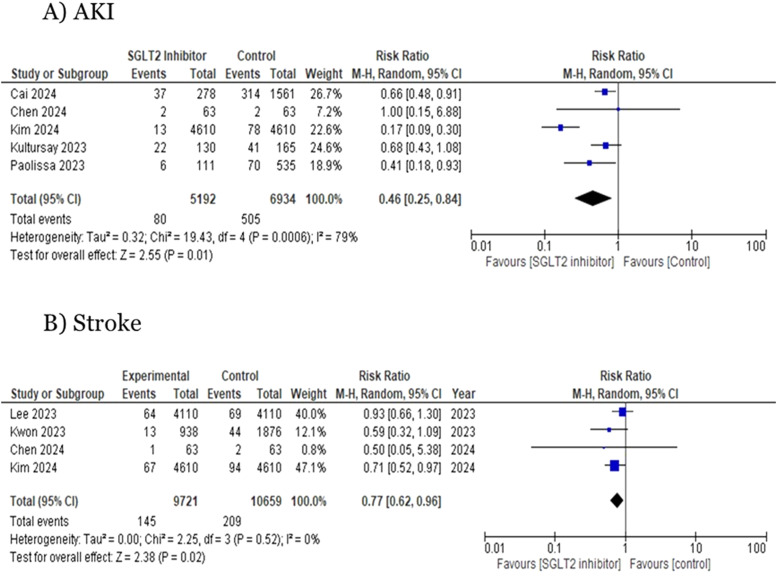
Forest plots for (A) AKI and (B) Stroke AKI: acute kidney injury.

Five studies reported data for AKI. The pooled analysis demonstrated that the SGLT2i plus PCI was associated with a significant decrease in the risk for AKI compared to no SGLT2i use (RR=0.46; 95 % CI = [0.25, 0.84]; *p* < 0.01; I^2^ =79 %, Fig. 4**A**). Owing to the high level of heterogeneity observed, a leave-one-out analysis was conducted, during which the study by Kim 2024 et al. was excluded. This exclusion substantially reduced the heterogeneity from 79 % to 0 % and the results remained significant (RR = 0.64, 95 % CI: 0.50 to 0.82, *p* < 0.01, I² = 0 %) (Figure S4).

#### 3.3.7 Stroke

Four studies reporting the risk of the incidence of stroke were evaluated. The SGLT2i plus PCI was associated with a significant decrease in the risk for stroke compared to no SGLT2i use (RR=0.77; 95 % CI=0.62, 0.96;*p* < 0.01;I^2^ =0 %, Fig. 4**B**).

## 4 Discussion

This comprehensive meta-analysis of 8 studies, incorporating a total of 24,229 type 2 diabetes patients with MI undergoing PCI, aimed to evaluate whether SGLT2i plus PCI was associated with improved clinical outcomes compared to PCI without SGLT2i use. In our investigation, we found that the use of SGLT2i was associated with a significant reduction in all-cause death, cardiovascular death, MACE and AKI. SGLT2i was also associated with a significantly reduced risk of HHF and stroke, compared to PCI without SGLT2i use. However, the risk of revascularization remained comparable across the two groups.

Our study complements existing research showing that while SGLT2i, primarily used to improve glycemic control in diabetic patients, also provides cardioprotective and nephroprotective effects [7,25,26]. Although the exact underlying mechanisms remain unclear, these effects may be associated with diuresis/natriuresis, enhanced kidney function, improved cardiac energy metabolism, induction of vasodilation, reduction in arterial blood pressure, reduced inflammation, reduced endothelial dysfunction and decreased arterial stiffness [10,[27], [28], [29]]. These effects were initially demonstrated in patients with heart failure (HF), which showed significant reductions in cardiovascular death, hospitalizations and renal events [9]. This prompted increased interest in exploring the potential benefits of SGLT2i in patients with AMI, with the EMMY trial being the first to investigate the efficacy and safety of an SGLT2i, empagliflozin, in patients with AMI undergoing PCI [30]. This trial reported a significantly decreased NT-proBNP concentration, a key predictor of cardiovascular events following MI, in the empagliflozin group, compared to placebo. Since then, various other trials and observational studies have investigated the impact of SGLT2i on clinical and echocardiographic outcomes, expanding upon these initial findings [[17], [18], [19], [20], [21], [22],^31^,^32^].

We observed a significant reduction in AKI associated with the use of SGLT2i. Following PCI, approximately 7 % of patients experience AKI, largely due to the use of contrast agents, defined as contrast-induced AKI (CI-AKI) [4]. CI-AKI is the third leading cause of hospital-acquired AKI, which is closely linked to in-hospital mortality [33]. Independent risk factors for AKI during PCI include severe baseline chronic kidney disease (CKD), cardiogenic shock, and ST-segment elevation myocardial infarction (STEMI) presentation [4]. Menne et al. also reported in their meta-analysis, that SGLT2i were associated with a reduced risk of AKI [34]. The exact mechanisms by which SGLT2i prevent AKI are not fully understood, especially given their association with hypovolemia, a key factor in acute prerenal failure. However, experimental studies suggest they may reduce tubular injury by increasing vascular endothelial growth factor (VEGF) and erythropoietin production and decreased peritubular inflammation and fibrosis [35]. Additionally, an important concept in the reno-protective function of SGLT2i is reduced hyperfiltration by increasing distal sodium delivery which leads to the stimulation of tubuloglomerular feedback, thereby reducing intraglomerular pressure [36]. Given the significant burden of CI-AKI and the protective properties of SGLT2i, investigating their integration into routine clinical practice is essential. The reduction in AKI could lead to improved clinical outcomes, including reduced hospital stay durations and decreased long-term kidney-related complications. In order to optimize their advantages in high-risk populations, future research should concentrate on establishing the optimal dosage and patient selection criteria. Results may also be improved by combining SGLT2i with other preventive measures like adequate hydration and minimizing contrast volume.

Our findings on all-cause and cardiovascular death align with those reported by Li et al., assessing the use of SGLT2i in improving cardiovascular outcomes in patients with diabetes mellitus (DM) who experienced AMI [37]. Animal studies have also shown SGLT2i to reduce mortality rates following MI by raising antioxidant levels and modifying cardiac metabolomes [38]. Moreover, these inhibitors also have been observed to reduce the size of infarctions, improve left ventricular function, and lessen the frequency of arrhythmias [39]. Such reductions in infarct size and improvements in ventricular function likely contribute to the reduced mortality and lower incidence of post-MI complications observed with these treatments.

A prospective study by Gamaza-Chulián et al. also demonstrated a reduction in left ventricular mass and improved global longitudinal strain in patients treated with SGLT2i compared to controls, indicating favorable structural and functional cardiac changes [40]. This evidence of reverse remodeling supports the idea that SGLT2i may not only prevent further damage but actively contribute to the repair of the myocardium, especially in patients with left ventricular hypertrophy or dysfunction. Our findings also indicate a significantly reduced risk of HHF. Previous research, including the EMPA-REG OUTCOME trial, reported significant reductions in hospitalizations in the SGLT2i, empagliflozin group [8]. The EMPACT-MI trial included 31.7 % of patients with type-2 diabetes who received empagliflozin treatment after MI [41]. PCI was performed in many of these patients. A subgroup analysis of this trial will provide additional insights into the efficacy of SGLT2i therapy in patients with type-2 diabetes and MI following PCI. Zelniker et al. also reported SGLT2i to be associated with a 23 % reduced risk of CV death and HHF, further contributing to the growing evidence of the cardioprotective role of SGLT2i [25]. SGLT2i also play a role in ketogenesis, which provides an alternative energy source for the myocardial cells in ischemic stress [42,43].

Our analysis reported non-significant findings in the risk of revascularization between the two groups. These results emphasize the need for additional research to clarify the role of SGLT2i in reducing revascularization risk, especially given their proven benefits in other cardiovascular outcomes. Revascularization, commonly used as an indicator of disease progression in patients with CV disease, may not be directly influenced by the mechanisms through which SGLT2i exert their benefits. Differences in clinical protocols between the included studies, such as the timing of follow-up assessments and variations in sample size, may have contributed to the significant heterogeneity observed in this outcome. Additionally, patient baseline characteristics such as the severity of underlying cardiovascular disease, presence of comorbidities, and particularly the use of different medications may also be responsible.

This is the largest meta-analysis to date that directly evaluated the clinical outcomes with the use of SGLT2i in patients with MI undergoing PCI. However, it is important to acknowledge that our study has some limitations, including observational study designs and varying patient demographics in our pooled studies which may contribute to heterogeneity in the results. Moreover, most of the included studies had relatively short follow-up durations, studies with longer follow-ups are required to evaluate the sustained impact of SGLT2i. Another limitation is the imbalance in sex representation among the included studies, the predominance of males over female participants could introduce a potential bias and limit the generalizability of our findings. High heterogeneity was observed in the occurrence of hospitalization due to HF, revascularization, and AKI. This heterogeneity was largely driven by Kim et al., who compared the efficacy of SGLT2 inhibitors with an active comparator, a dipeptidyl peptidase-4 (DPP-4) inhibitor. Furthermore, Paolisso et al., stratified the groups according to the use of SGLT2 inhibitors and other oral antidiabetics (OAD). Although the outcomes remained significant after removing each study, the differences in the choice of comparators may explain the high heterogeneity. Furthermore, we were unable to categorize the most potent type of SGLT2 inhibitor across all outcomes due to the lack of studies reporting this information. Therefore, future studies are warranted to conduct head-to-head comparisons of different types of SGLT2 inhibitors and their efficacy compared to other OADs. Some observational studies included patients who were already on SGLT2i therapy before PCI and continued it afterward, while others included patients who started SGLT2i therapy only after undergoing PCI. This could have led to differences in observed clinical outcomes. We therefore recommend future randomized control studies to assess the long-term benefits of SGLT2i combined with PCI.

## 5 Conclusion

In conclusion, this meta-analysis demonstrates that SGLT2i plus PCI in patients with MI and type 2 diabetes is associated with improved cardio-renal outcomes. Future large-scale randomized controlled trials are needed to better understand the long-term clinical benefits of SGLT2i in this patient population.

## Ethical approval

No ethical approval was required for the study.

## Consent

No consent was needed.

## CRediT authorship contribution statement

**Huzaifa Ul Haq Ansari:** Writing – original draft, Supervision, Methodology, Data curation, Conceptualization. **Muhammad Ammar Samad:** Writing – original draft, Project administration, Methodology, Data curation, Conceptualization. **Eman Mahboob:** Writing – original draft, Visualization, Validation, Supervision, Project administration. **Eeshal Zulfiqar:** Writing – original draft, Methodology. **Shurjeel Uddin Qazi:** Formal analysis, Data curation. **Areeba Ahsan:** Writing – original draft, Methodology, Data curation. **Mushood Ahmed:** Writing – review & editing, Writing – original draft, Supervision. **Faizan Ahmed:** Writing – review & editing. **Raheel Ahmed:** Writing – review & editing, Supervision. **Shafaqat Ali:** Writing – review & editing, Supervision. **Mahboob Alam:** Writing – review & editing, Visualization, Validation. **Jamal S. Rana:** Writing – review & editing, Visualization, Validation, Supervision, Conceptualization. **Gregg C. Fonarow:** Writing – review & editing, Visualization, Validation, Supervision, Conceptualization.

## Declaration of competing interest

The authors declare that they have no known competing financial interests or personal relationships that could have appeared to influence the work reported in this paper.

## Data Availability

All data generated or analyzed during this study are included in this article. Further inquiries can be directed to the corresponding author.

## Appendix Supplementary materials

Image, application 1
