# Supplementary material for: Sodium-glucose cotransporter 2 inhibitors in patients with type 2 diabetes and myocardial infarction undergoing percutaneous coronary intervention: A systematic review and meta-analysis
Source: Am J Prev Cardiol. 2024 Dec 31;21:100927. doi: 10.1016/j.ajpc.2024.100927 (PMC11757226; doi:10.1016/j.ajpc.2024.100927)
Supplement: Supplementary file 1 [file mmc1.docx]

**Supplementary Appendix**

| **Database** | **Search string** | **Records** |
| --- | --- | --- |
| PubMed/MEDLINE | (("Sodium-glucose"[All Fields] AND ("symporters"[MeSH Terms] OR "symporters"[All Fields] OR ("co"[All Fields] AND "transporter"[All Fields]) OR "co transporter"[All Fields]) AND "inhibitor-2"[All Fields]) OR ("sodium glucose transporter 2 inhibitors"[Pharmacological Action] OR "sodium glucose transporter 2 inhibitors"[MeSH Terms] OR "sodium glucose transporter 2 inhibitors"[All Fields] OR ("sglt2"[All Fields] AND "inhibitors"[All Fields]) OR "sglt2 inhibitors"[All Fields]) OR ("empagliflozin"[Supplementary Concept] OR "empagliflozin"[All Fields]) OR ("dapagliflozin"[Supplementary Concept] OR "dapagliflozin"[All Fields] OR "dapagliflozin s"[All Fields]) OR ("canagliflozin"[MeSH Terms] OR "canagliflozin"[All Fields]) OR ("bexagliflozin"[Supplementary Concept] OR "bexagliflozin"[All Fields])) AND ("myocardial infarction"[MeSH Terms] OR ("myocardial"[All Fields] AND "infarction"[All Fields]) OR "myocardial infarction"[All Fields] OR ("microbiology"[MeSH Subheading] OR "microbiology"[All Fields] OR "mi"[All Fields])) AND ("percutaneous coronary intervention"[MeSH Terms] OR ("percutaneous"[All Fields] AND "coronary"[All Fields] AND "intervention"[All Fields]) OR "percutaneous coronary intervention"[All Fields] OR "PCI"[All Fields]) | 63 |
| Scopus | (TITLE-ABS-KEY("Sodium-glucose co-transporter inhibitor-2" OR "SGLT2 inhibitors" OR "empagliflozin" OR "dapagliflozin" OR "canagliflozin" OR "bexagliflozin"))  AND  (TITLE-ABS-KEY("myocardial infarction" OR "MI"))  AND  (TITLE-ABS-KEY("percutaneous coronary intervention" OR "PCI")) | 163 |
| Embase | ('sodium glucose co transporter 2 inhibitor'/exp OR 'sodium glucose co transporter 2 inhibitor' OR 'sglt2 inhibitor' OR 'empagliflozin'/exp OR empagliflozin OR 'dapagliflozin'/exp OR dapagliflozin OR 'canagliflozin'/exp OR canagliflozin OR 'bexagliflozin')  AND  ('myocardial infarction'/exp OR 'myocardial infarction' OR 'mi')  AND  ('percutaneous coronary intervention'/exp OR 'percutaneous coronary intervention' OR 'pci') | 365 |
| Cochrane Library | ("Sodium-glucose co-transporter inhibitor-2" OR "SGLT2 inhibitors" OR "empagliflozin" OR "dapagliflozin" OR "canagliflozin" OR "bexagliflozin")  AND  ("myocardial infarction" OR "MI")  AND  ("percutaneous coronary intervention" OR "PCI") | 53 |

Table S2: Details of MACE definitions used in each study

| No. | Study name | MACE components |
| --- | --- | --- |
| 1. | Kwon et al. | composite of all‐cause death, nonfatal MI, and nonfatal ischemic stroke |
| 2. | Paolisso et al. | cardiovascular death, recurrent AMI, and hospitalization for HF |
| 3. | Lyu et al. | all-cause mortality, non-fatal MI (NFMI), revascularization, cerebrovascular accident, and rehospitalization |
| 4. | Chen et al. | myocardial infarction, is-chemic stroke, and death from cardiovascular causes |
| 5. | Kim et al. | repeat revascularization, MI, stroke, HF, all-cause death and end stage renal disease |

Figure S1: Risk of bias summary for included observational studies


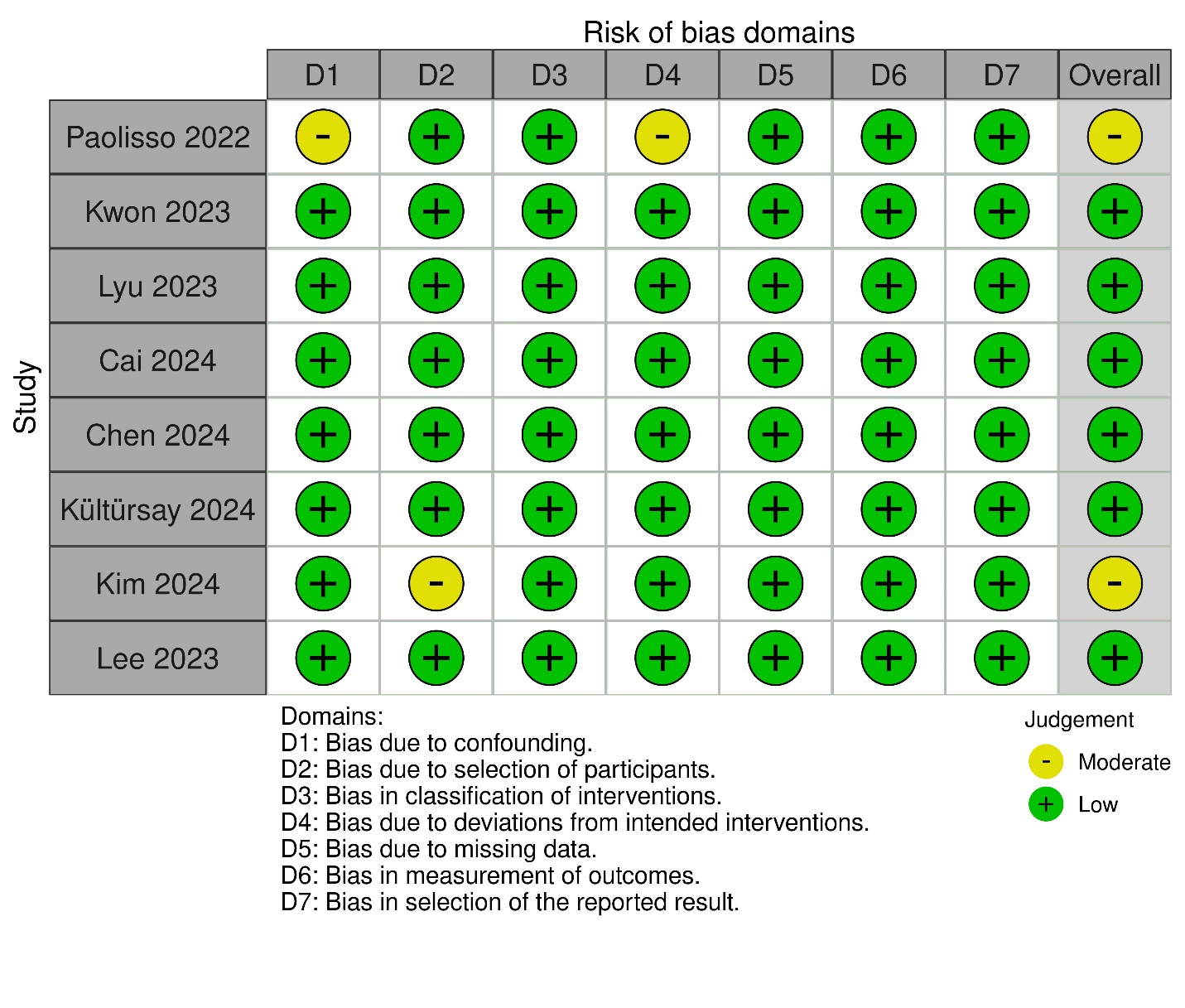


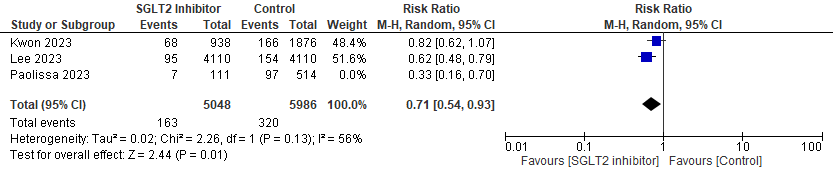


Figure S2: Sensitivity analysis for hospitalization due to heart failure outcome


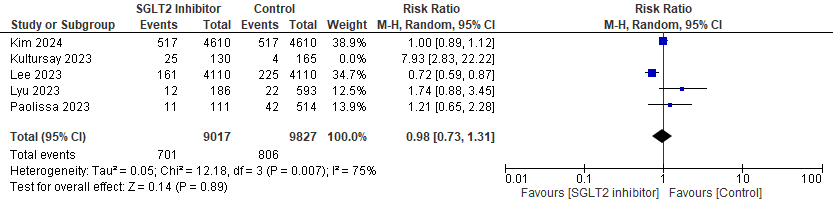


Figure S3: Sensitivity analysis for revascularization outcome


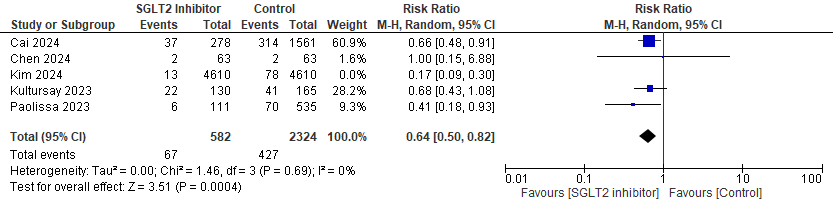


Figure S4: Sensitivity analysis for acute kidney injury outcome
